# Antibacterial small molecules targeting the conserved TOPRIM domain of DNA gyrase

**DOI:** 10.1371/journal.pone.0180965

**Published:** 2017-07-10

**Authors:** Scott S. Walker, Marc Labroli, Ronald E. Painter, Judyann Wiltsie, Brad Sherborne, Nicholas Murgolo, Xinwei Sher, Paul Mann, Paul Zuck, Charles G. Garlisi, Jing Su, Stacia Kargman, Li Xiao, Giovanna Scapin, Scott Salowe, Kristine Devito, Payal Sheth, Nichole Buist, Christopher M. Tan, Todd A. Black, Terry Roemer

**Affiliations:** 1 Merck & Co., Inc., Kenilworth, New Jersey, United States of America; 2 Merck & Co., Inc., West Point, Pennsylvania, United States of America; 3 Merck & Co., Inc., Boston, Massachusetts, United States of America; Purdue University, UNITED STATES

## Abstract

To combat the threat of antibiotic-resistant Gram-negative bacteria, novel agents that circumvent established resistance mechanisms are urgently needed. Our approach was to focus first on identifying bioactive small molecules followed by chemical lead prioritization and target identification. Within this annotated library of bioactives, we identified a small molecule with activity against efflux-deficient *Escherichia coli* and other sensitized Gram-negatives. Further studies suggested that this compound inhibited DNA replication and selection for resistance identified mutations in a subunit of *E*. *coli* DNA gyrase, a type II topoisomerase. Our initial compound demonstrated weak inhibition of DNA gyrase activity while optimized compounds demonstrated significantly improved inhibition of *E*. *coli* and *Pseudomonas aeruginosa* DNA gyrase and caused cleaved complex stabilization, a hallmark of certain bactericidal DNA gyrase inhibitors. Amino acid substitutions conferring resistance to this new class of DNA gyrase inhibitors reside exclusively in the TOPRIM domain of GyrB and are not associated with resistance to the fluoroquinolones, suggesting a novel binding site for a gyrase inhibitor.

## Introduction

Antibiotic resistance is an immediate and growing danger to human health. The morbidity and mortality wrought by these resistant bacteria know no international or socioeconomic boundaries. The so-called ESKAPE pathogens typify the risks and experience with controlling and combating bacterial infections in the community and in the clinic [1]. Amongst the ESKAPE pathogens are the Gram-negatives *Klebsiella pneumoniae*, *Acinetobacter baumannii*, *Pseudomonas aeruginosa* and *Escherichia coli*. Historically, these Gram-negative pathogens have been controlled with many of the currently approved antimicrobials. With the emergence and transmissibilityof a number of resistance mechanisms, such as β-lactamases, efflux pumps, or drug-modifying enzymes, treatment options can be very limited or non-existent [2]. These life-threatening, difficult-to-treat infections have prompted a call for the identification and development of novel antibiotics addressing new modalities or new chemistry that circumvents resistance to current therapies [3].

The fluoroquinolones (FQs, e.g, ciprofloxacin) are an essential class of antibiotics encompassing several generations of modification and improvement. Ciprofloxacin, for example carries a large number of approved therapeutic indications including urinary tract, respiratory tract, skin and skin structure infections, among others (fda.gov). There are however significant limitations to the use of this important class of antibiotics [4, 5]. Bacterial DNA gyrase, the target of the FQs, is a heterotetrameric complex of approximately 380 kDa containing two A (GyrA) and two B (GyrB) subunits that controls the torsional strain induced by the positive supercoils resulting from transcription and DNA replication [6, 7]. By creating a double strand break (gate segment) and passing another strand of DNA (transfer segment) through the break and resealing it in an ATP-dependent reaction, gyrase introduces negative supercoils, thereby reducing positive supercoiling [8]. That reaction, the structure of gyrase, and the effects of inhibitors such as the FQs have been studied and reviewed in considerable detail (e.g., [9–11]). Gyrase is a clinically validated, structurally tractable target for antibacterial drug development, and in recent years a considerable number of non-FQ gyrase inhibitors have been described and reviewed [12, 13]. Bacterial topoisomerase II inhibitors can be divided into two broad categories; those that inhibit the essential ATPase function of the B subunit and those that inhibit gyrase in a different manner. The founding member of the ATPase inhibitor class is novobiocin, an aminocoumarin natural product, while a number of other inhibitors have also been developed that target the ATPase domain [13]. Gyrase inhibitors can also act through interactions with other regions of the tetrameric complex. Examples of an inhibitor class that differs from ATPase inhibitors and FQs are NXL101 and GSK299423 [14, 15]. An important region in the GyrB subunit is the TOPRIM domain; a conserved structural element found in a number of DNA-interacting enzymes such as topoisomerases (types IA/II), DnaG-like primases, archael nucleases, and RecR-like DNA repair proteins [16]. AZD0914, a novel gyrase and topoisomerase IV inhibitor appears to interact with the TOPRIM domain [17, 18].

As an alternative to target-focused approaches to drug discovery [19, 20] we began with a collection of proprietary small molecules that had demonstrated activity against *E*. *coli* lacking efflux capability (Δ*tolC*) and having reduced barrier function (lipopolysaccharide) due to a mutation in *lpxC*. This strain enabled the identification of molecules that would likely otherwise not be expected to inhibit growth of wild-type bacteria, but may yield novel target/compound pairs for further development [21]. The collection of bioactives was then counter-screened for activity against a model eukaryote (*Saccharomyces cerevisiae*) to eliminate generally bioactive molecules and examined for suitability for synthesis and derivatization as lead molecules for drug development. Further triage of this set encompassed testing for cell lysis in bacteria and erythrocytes [22, 23], excluding those with activity close to the antibacterial activity. As the annotated library of bioactives became more refined, we sought to identify the pathway involved through inhibition of macromolecule synthesis. Finally, we attempted target identification through selection for resistance and whole genome sequencing and, where possible, biochemical assays. From this screening and evaluation effort, we detail here the discovery of a novel class of Gram-negative bacterial DNA gyrase inhibitors targeting the TOPRIM domain.

## Materials and methods

### Strains

**Fig 1:**
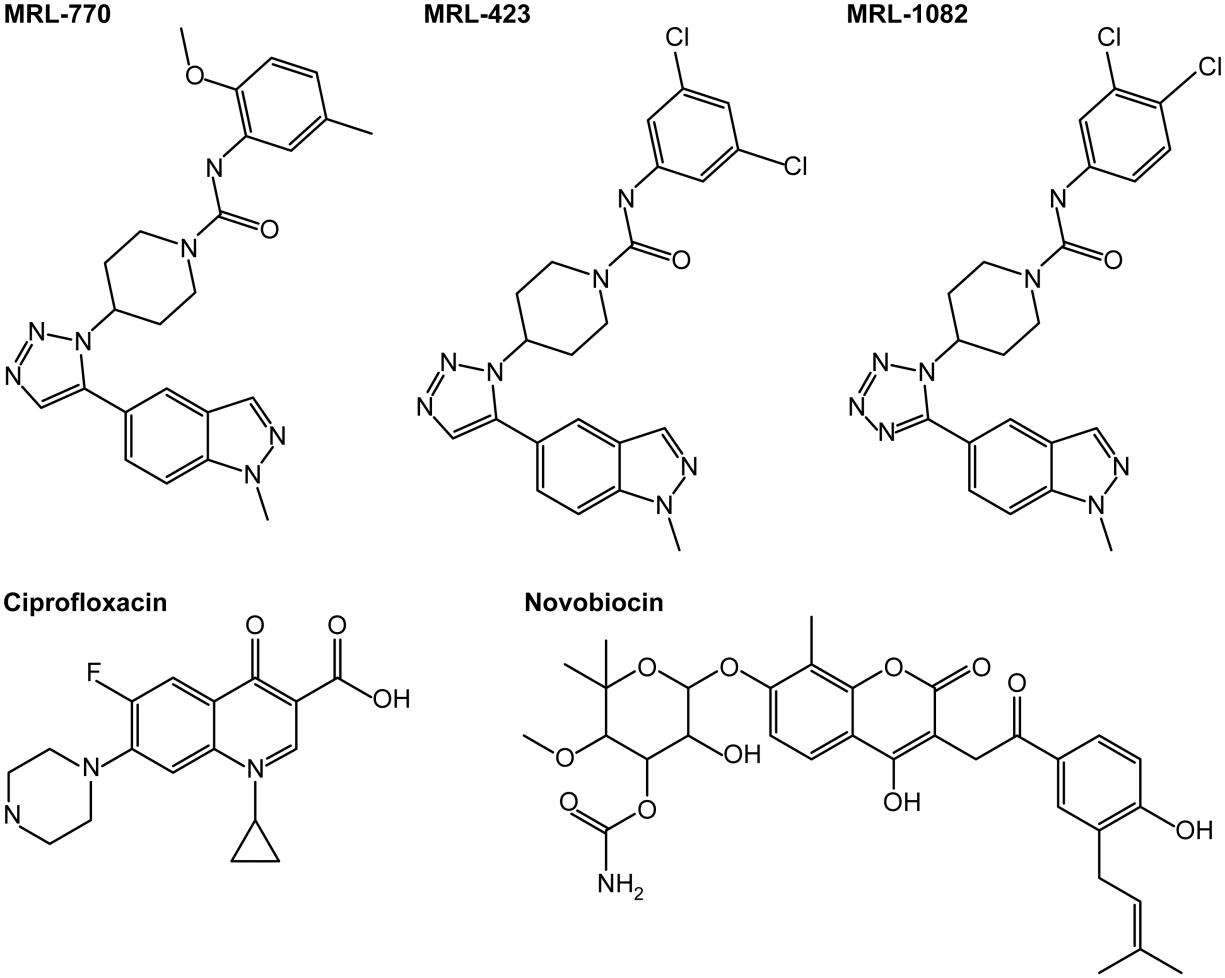
Chemical structures of novel DNA gyrase inhibitors and reference compounds.

*E*. *coli* HS151 (Δ*tolC*) has been described previously [24]. Gyrase inhibitor-resistant isolates (GYR101-108) were derived by selection for spontaneous resistance to MRL-770 or MRL-423 (Fig 1) in *E*. *coli* HS151 as described below. *E*. *coli* BW25113 and the Keio gene deletion collection based on BW25113 [25] were obtained from Thermo Fisher Scientific (Waltham, MA) and described in [26]. The plasmid-borne FQ resistance determinant, *qnrVC3* [27], was kindly provided by D. Hooper (Massachusetts General Hospital). MB9796 (*A*. *baumannii*) is a derivative of MB9797 (ATCC19606) lacking lipopolysaccharide [28]. The efflux-defective *P*. *aeruginosa* strain MB5890 (CB398) is a derivative of MB5919 (PAO1) [29]. MB2884 (*E*. *coli*) and CL15245 (*K*. *pneumoniae*) are clinical isolates from the Merck & Co., Inc., Kenilworth, NJ, USA, strain collection. *S*. *aureus* COL is a methicillin-resistant clinical isolate [30, 31].

### Macromolecule labeling

Macromolecule synthesis profiling was conducted as described by Montgomery, et al. [32] with minor modifications. *E*. *coli* strain JL553 (*tolC*, *lysE*) [32] was used to measure inhibition of DNA, RNA, protein, peptidoglycan and phospholipid biosynthesis while *E*. *coli* MB5500 (*lpxC* [*envA1*], *galE*; Merck & Co., Inc., Kenilworth, NJ, USA, strain collection) was used to measure inhibition of lipopolysaccharide biosynthesis. Radioisotope labeled precursors were used at the following final concentrations [2-^14^C]-thymidine (2.5 μCi/mL), [^14^C(U)]-uridine (0.5 μCi/mL), L-[4, 5-^3^H]-leucine (5 μCi/mL), [2-^3^H]-glycerol (2 μCi/mL), [^3^H]-diaminopimelic acid (3 μCi/mL), D-[1-^14^C]-galactose (0.5 μCi/mL). Compound concentrations (3-fold serial dilutions) were chosen to place the minimum inhibitory concentration (see below) near the center of the dose range. Labeled cultures were incubated with compound for 25 min; stopped with trichloroacetic acid, filtered and incorporation was measured as described [32]. Percent inhibition of incorporation was calculated relative to an untreated control culture for each labeled precursor and plotted with GraphPad Prism.

### Agarose gel DNA gyrase assays

Conditions to measure the supercoiling of a relaxed, closed-circular plasmid substrate and cleavage complex stabilization in agarose gels were adapted from the literature and carried out as described below [33]. Plasmid supercoiling reactions were assembled by combining 0.4 μL compound (in 100% DMSO), 12.5 μL H_2_O, 4 μL 5Xbuffer, 0.4 μL relaxed pUC19 DNA substrate (0.5 μg), 1 mL dilute DNA gyrase (buffer, substrate, and enzyme from New England Biolabs, Beverly, MA). Alternatively the substrate pHOT-1 (TopoGEN, Buena Vista, CO) was used as noted (the substrate from New England Biolabs has been discontinued). *P*. *aeruginosa* DNA gyrase was supplied by Inspiralis, Norwich, UK. Reactions were allowed to proceed for 30 min. at 37°C after which 5 μL of stop buffer (5% sarkosyl, 25% glycerol, 1X Blue Juice [Invitrogen, Carlsbad, CA]) were added. Reactions were then extracted with 20 μL chloroform/isoamylOH (24:1) and a portion of the aqueous phase was loaded into a well in a 1% agarose/Tris-borate EDTA gel. Electrophoresis was conducted at 35 VDC until the loading dye migrated about 6.5 cm down the gel. DNA in the gel was then stained in 0.5 μg/mL ethidium bromide in water for 30 min. and rinsed (3X) in H_2_O. The gel was illuminated with UV light, photographed, and DNA quantitated with an Alpha Imager and accompanying software (ProteinSimple, San Jose, CA). Following background subtraction, image data were used to estimate the concentration to achieve 50% inhibition (IC_50_) on a four-parameter curve fit in GraphPad Prism software (IC_50_ derived from 2–3 independent experiments).

Cleavage complex stabilization was detected by assembling a gyrase reaction, as above, containing 0.4μL compound (in 100% DMSO), 12.5 μL H_2_O, 4 μL 5X buffer, 0.4 μL supercoiled pUC19 DNA substrate (0.4 μg), 3 μL gyrase (15 U). The reaction was incubated at 25°C for 60 min. after which 3 μL 2% SDS and 3 μL 1 mg/mL proteinase K were added. After 30 min. at 37°C, 7 μL 5X loading dye (25% glycerol, 1X Blue Juice) were added and 15 μL of each reaction were then loaded onto 1% agarose/tris-borate EDTA and ethidium bromide (0.5 μg/mL) gel and run at 50-65VDC until loading dye migrated about 6.5 cm down the gel. DNA in the gel was illuminated with UV light and photographed as above.

### Expression and purification of recombinant *E*. *coli* DNA GyrA and GyrB

The *E*. *coli gyrA* and *gyrB* (wild-type and t1537c (F513L)) genes with a N-terminal His6 tag sequence followed by Tobacco Etch Virus (TEV) cleavage site (MGSSHHHHHHSSGENLYFQGMS) were cloned into pET28(+) vector (EMD Millipore). The recombinant NHisS6TEV gyrase subunit protein (GyrA or B) was expressed in *E*. *coli* strain BL21(DE3) RIL in Luria Broth supplemented with 50 μg/mL kanamycin for 4 hours at 37°C with 0.5 mM IPTG. The cells were harvested by centrifugation for 15 minutes at 6000 × g. Pellets were then resuspended in 20 mM Tris-HCl, 0.8 M NaCl, 10% glycerol, 2 mM β-mercaptoethanol, 30 mM imidazole, and 1 mg/mL protease inhibitor cocktail III at pH 7.9 and then lysed with a microfluidizer. The cell lysate was clarified by centrifugation at 100,000 × g for 1 hour at 4°C. The supernatant was filtered and loaded onto a Ni^2+^-IMAC column (Qiagen, Netherlands) equilibrated with 20 mM Tris-HCl (pH = 7.9), 0.8 M NaCl, 10% glycerol, 2 mM β-mercaptoethanol, 30 mM imidazole. The protein was eluted using an imidazole gradient (30 mM to 500 mM) containing 20 mM Tris-HCl (pH = 7.9), 25mM NaCl, 10% glycerol, and 2 mM β-mercaptoethanol. Fractions containing gyrase subunit protein (as monitored by sodium dodecyl sulfate polyacrylamide gel electrophoresis (SDS-PAGE)) were pooled and loaded onto a Q-Sepharose column (GE Healthcare, Princeton, NJ, USA) equilibrated in 20 mM Tris-HCl (pH = 7.9), 25mM NaCl, 10% glycerol, and 2 mM β-mercaptoethanol. The protein was eluted using a salt gradient (25 mM to 500 mM) and fractions corresponding to purified gyrase subunit protein were pooled and dialyzed in 20 mM Tris (pH = 7.9), 400 mM NaCl, 10% glycerol, and 2 mM β-mercaptoethanol with 1:20 molar ratio TEV protease. The cleaved protein and TEV protease solution was applied over Ni^2+^IMAC column (Qiagen, Netherlands) to separate HIS-tagged TEV. Gyrase subunit protein from the follow-through was concentrated for final purification step by size exclusion chromatography using a Superdex 200 26/60 column (GE Healthcare, Princeton, NJ, USA) equilibrated in 20 mM Tris-HCl (pH = 7.9), 0.5 M KCl, 10% glycerol, 2 mM β-mercaptoethanol. Fractions with >95% pure protein were collected and concentrated to 2.5 mg/mL (GyrA) and 0.5 mg/mL (GyrB; WT/F513L) using a centrifugal concentrator. The final storage buffer was 20 mM Tris-HCl (pH = 7.9), 0.5 M KCl, 50% glycerol, 2 mM β-mercaptoethanol. The identity of GyrB (WT/F513L) and GyrA proteins were confirmed by electrospray ion trap mass spectrometry (ESI-Ion-Trap-MS) using a LTQ-XL mass spectrometer (ThermoScientific, Rockford, IL, USA) and the Xcalibur software platform (ThermoScientific). *E*. *coli* gyrase holoenzyme was reconstituted at 1 μM with equimolar amounts of GyrA and GyrB subunits for 30 min on ice in buffer containing 50 mM Tris-HCl (pH = 8), 100 mM KCl, 5% glycerol, 2 mM DTT.

### Broth microdilution minimum inhibitory concentration determination

Compound doubling dilutions were prepared in 100% DMSO (Sigma Aldrich) and 2 μL of each dilution was transferred to a 96-well plate with lid (Thermo Scientific). Inoculum for all MIC testing was prepared using the BBL Prompt Inoculation System (Becton Dickinson, Franklin Lakes, NJ USA) as described by the manufacturer to produce a suspension of cells at approximately 1.5 x 10^8^ colony forming units (CFU)/mL [34–36] and a further dilution (1:1000) was made into cation adjusted Mueller-Hinton broth (CAMH broth, Becton-Dickinson). One hundred μL of this inoculum were then transferred to each well of the 96-well plate containing diluted compound (above). Plates were covered and incubated overnight (18 hrs.) at 37°C. The MIC for the compound was determined as the minimum concentration required to completely inhibit visible growth of the cells.

### In vitro time-dependent bactericidal studies

A culture of *E*. *coli* HS151 in CAMH broth was grown overnight (18 hrs.) at 37°C, diluted 1:10,000 in 200 mL of fresh CAMH broth and further incubated for 1 hour at 37°C with shaking. A time-zero sample was then removed and 20 mL portions of the remaining culture were transferred into 125 mL vented flasks to which were added MRL-1082 or control antibiotics, at the indicated concentrations. These flasks were then incubated at 37°C with shaking (70 rpm). Samples were withdrawn at the indicated times, plated on blood agar (trypticase soy agar with 5% sheep blood, Becton-Dickinson) and, following overnight growth at 37°C, colonies were counted to determine CFU/mL.

### Resistance selection and whole genome sequencing

The MRL-770 or MRL-423 agar-dilution MIC was determined prior to selection for resistance by first serially diluting the compound in 100% DMSO (Sigma Aldrich) followed by the addition of the diluted compound to 4 mL of molten CAMH agar in each chamber of an 8-well dish (Thermo Scientific). After the agar solidified, 5 μL of a saturated bacterial culture, grown in CAMH broth, were dispensed on the agar surface and allowed to dry at room temperature. The plate was then incubated overnight at 37°C and the agar MIC was identified as the lowest dilution of compound that completely inhibited visible growth. To select for resistance, a 100 mm petri dish was filled with 25 mL CAMH agar containing 8 μg/mL MRL-770 or 0.5 μg/mL MRL-423 (Fig 1). The cells in 1 mL of an overnight culture of *E*. *coli* HS151 (~10^9^ CFU/mL) were concentrated by centrifugation and spread onto the surface of an MRL-770 or MRL-423 containing plate. After the surface dried at room temperature the plate was incubated at 37°C overnight. Any colonies that arose were counted and an approximate frequency of resistance was calculated after confirming resistance by restreaking on media containg compound as well as measuring MICs (see Results). Genomic DNA from the parent strain (*E*. *coli* HS151) and purified (restreaked) resistant isolates was obtained with a GeneJET genomic DNA purification kit according to the manufacturer’s instructions (Fisher Scientific). Assembled whole genome mutant sequences were then compared to the parent strain and polymorphisms were identified as described [37–40].

### *yacG* deletion

The *yacG* deletion allele from the Keio collection [26] was amplified by PCR (primers: GGACATAAGAGCCGTTTTGCCATTCG and CAGGCCTAAAGGGATTTCTAACTCATTGAT) and moved to BW25113 Δ*tolC* as described below. The *E*. *coli* strain BW25113 Δ*tolC* containing pKD46 [25] was used to inoculate SOB (Invitrogen)/0.2% w/v arabinose (Sigma Aldrich)/ampicillin (100 μg/mL) and grown overnight at 30°C. The overnight culture was then diluted 1/40 into 20 mL fresh SOB/0.2% w/v arabinose/ampicillin (100 μg/mL) and grown at 30°C while shaking for ~3 hrs until OD_600_ = 0.5. Cells were pelleted by centrifugation at 4000 rpm for 10 min, washed with 40 mL ice cold sterile, distilled H_2_O, then 3 x 1 mL ice cold 10% glycerol followed by centrifugation at 5000 g for 3 minutes. The pellet was resuspended in 100 μL of 10% glycerol (1/200 of starting culture). For transformation, 5 μL of gel purified PCR product was added to 50 μL cells, mixed, and immediately electroporated in a 0.2 cm gap cuvette at 2.5kV, 25 μF, 200 Ω (BioRad, Hercules, CA USA). Electroporated cells were recovered in 1 mL SOC (Invitrogen)/0.2% arabinose at 37°C for 2 hours with shaking. Cells in amounts of 50 and 950 μL were then plated on LB agar supplemented with kanamycin (25 μg/mL) and incubated overnight at 37°C. Correct gene replacements were verified by PCR.

## Results

**Fig 2:**
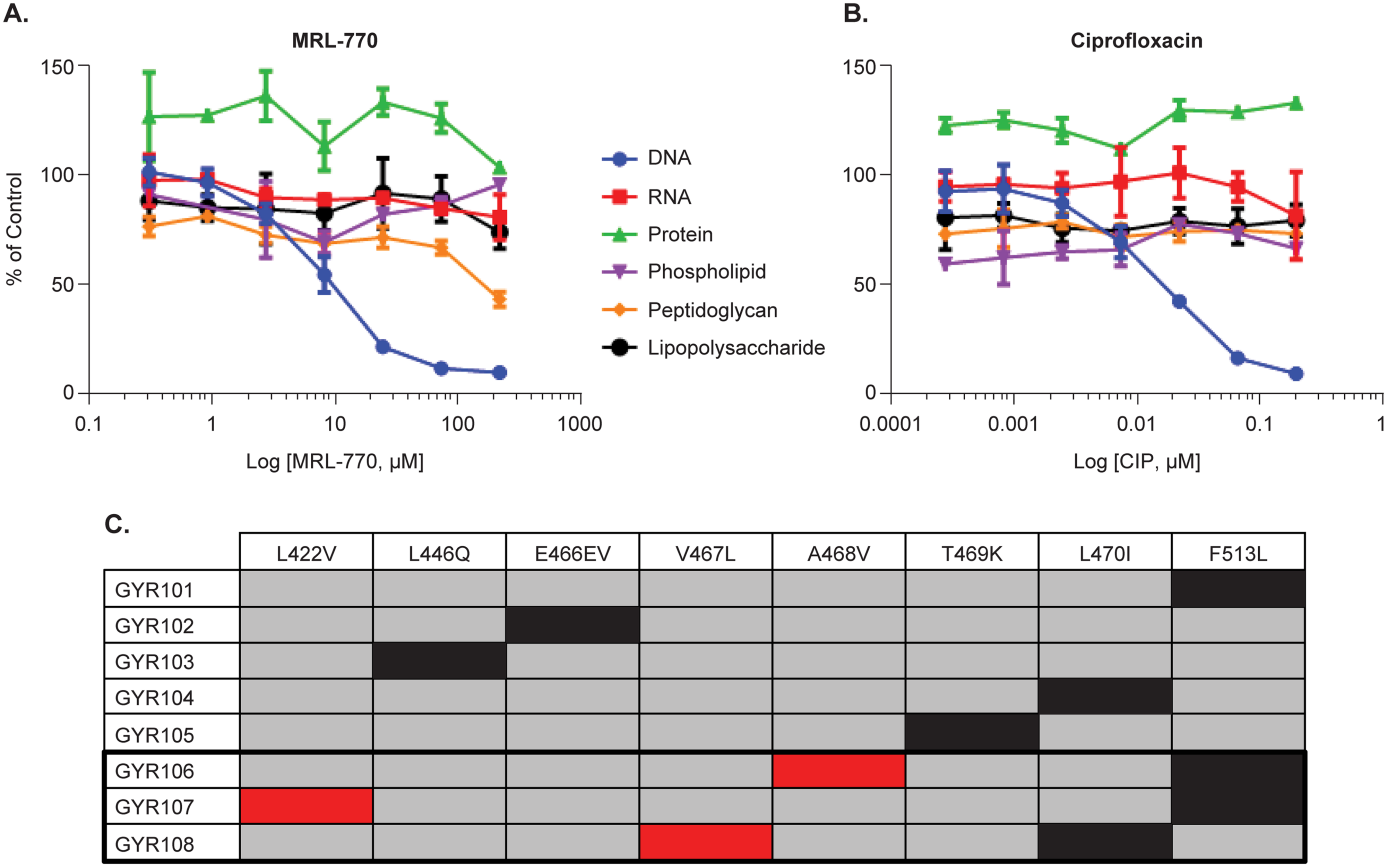
Inhibition of macromolecule synthesis in *E*. *coli*. (A) Dose-dependent, selective inhibition of DNA synthesis by MRL-770 in *E*. *coli* JL553. (B) Dose-dependent, selective inhibition of DNA synthesis by ciprofloxacin. (C) Schematic representation of the *E*. *coli* GyrB mutations conferring resistance to MRL-770 series compounds. For each mutant listed in the first column a black-filled cell identifies the mutation site and amino acid substitution (top row). GYR106-108 mutants were isolated following reselection for higher-level resistance to MRL-423. Red cells designate second site amino acid location and substitution acquired in the reselected mutants.

Beginning with a collection of compounds active against a sensitized *E*. *coli* strain containing mutations affecting the integrity of the outer membrane and a xenobiotic efflux system, we sought to categorize these bioactives by inhibition of macromolecule synthesis. One such compound (MRL-770, Fig 1) exhibited selective and reproducible inhibition of DNA synthesis in *E*. *coli* JL553 (Fig 2). This inhibition, observed following a brief exposure to the compound (25 min), began at concentrations near the minimum inhibitory concentration (MIC; 4.5 μM; 2 μg/mL) and produced a dose-response profile similar to the control antibiotic ciprofloxacin. Additional in vitro microbial susceptibility testing shows that MRL-770 is active against sensitized strains of bacteria, but as Table 1 indicates efflux or cell permeability may be a major impediment to broader Gram-negative antibacterial potency.

**Table 1:** Antibacterial activity of novel gyrase inhibitors.

Strain name	Description	MIC (μg/mL)							
		MRL-770	MRL-423	MRL-1082	ciprofloxacin	levofloxacin	novobiocin	rifampicin	tetracycline
HS151	*E*. *coli* ΔtolC	2	0.25	0.0625	0.00195	0.00195	2	8	0.5
MB2884	*E*. *coli*	>64	>64	>64	0.007813	0.0156	>64	8	1
MB9796	*A*. *baumannii* Δ*lpxC*	4	0.5	0.5	0.0625	0.0156	<0.0625	<0.0625	0.25
MB9797	*A*. *baumanii*	>64	>64	>64	>0.125	0.125	8	2	2
MB5890	*P*. *aeruginosa*(effluxΔ)	4	1	0.25	0.00195	0.0039	64	32	0.25
MB5919	*P*. *aeruginosa*	>64	>64	>64	0.125	>0.125	64	32	32
CL15245	*K*. *pneumoniae*	>64	>64	>64	0.0156	0.0156	>64	32	2
COL	*S*. *aureus* (MRSA)	>64	>64	>64	>0.125	>0.125	0.125	<0.0625	>64

To understand the mechanism of growth inhibition by our initial hit, MRL-770, spontaneous resistance was selected for in the efflux-deficient *E*. *coli* strain HS151. Following 24–48 hours on agar plates containing 8 μg/mL (4XMIC) rare resistant isolates were found at a rate of approximately 5X10^-9^. Comparison of the genome sequences of resistant mutants with that of the wild-type parent strain identified missense mutations and a single codon insertion mutation in *gyrB* (DNA gyrase) that were associated with selective resistance to MRL-770 (Fig 1, Table 2). The more potent analogs MRL-423 and MRL-1082 (Fig 1, Table 2) were designed and synthesized as part of a compound optimization program based on our initial discovery of MRL-770 and confirmation of the target. The relative location of each mutation, all of which reside within the conserved TOPRIM domain of GyrB [16], is depicted graphically in Fig 2C. Interestingly the single amino acid substitution mutants are not cross-resistant to other DNA gyrase inhibitors such as fluorquinolones and novobiocin, nor do they show any changes in susceptibility to other antibiotics (Table 2). The unusual codon insert mutant, GyrB (E466EV) has a 4-8-fold higher MIC to FQs. In an X-ray crystal structure of *S*. *aureus* DNA gyrase, the equivalent residue in GyrB (E477) is adjacent to a QRDR loop [14] and a codon insert at that site could affect positioning of the QRDR loop and thereby affect FQ binding (see Discussion). A mutation residing in the hotspot region of GyrA conferring moderate resistance to FQs does not affect sensitivity to our novel compounds (Fig 1 and Table 2). Selection for spontaneous resistance to MRL-423 identified a single isolate (frequency of resistance = 3.4 x 10^−10^) with an amino acid substitution mutation in GyrB (T469K) that was not resistant to MRL-770. Aside from GyrB (E466EV), resistance to MRL-423 was 4 to 16-fold higher than the parent strain *E*. *coli* HS151, suggesting that selection for higher level resistance to MRL-423 may provide additional insights into the cellular target(s). Reselection for resistance to MRL-423 in either the GyrB (F513L) or GyrB (L470I) mutant background identified additional rare spontaneous mutations in the same region of GyrB (Table 2, Fig 2C).

**Table 2:** Antibacterial susceptibility of *E*. *coli gyrB* mutants.

Strain name	GyrB mutation	MIC (μg/mL)								
		MRL-770	MRL-423	MRL-1082	Cipro-floxacin	Levo-floxacin	Novo-biocin	Rif-ampicin	Trimeth-oprim	Tetra-cycline
HS151	none	2	0.25	0.0625	0.00195	0.0039	2	8	0.0625	0.5
GYR101	F513L	>64	2	>64	0.00195	0.0078	2	8	0.0625	0.5
GYR102	E466EV	>64	>64	8	0.0078	0.03125	1	8	0.0625	0.5
GYR103	L446Q	>64	0.5	0.0625	0.0039	0.0078	2	8	0.0625	0.5
GYR104	L470I	>64	1	0.25	0.00195	0.0078	2	8	0.0625	0.5
GYR105	T469K	1	1	0.25	0.00195	0.0039	2	8	0.0625	0.5
GYR106	F513L/A468V	>64	>64	>64	0.00195	0.0078	2	8	0.0625	0.5
GYR107	F513L/L422V	>64	>64	>64	0.00195	0.0039	2	8	0.0625	0.5
GYR108	L470I/V467L	>64	>64	2	0.0039	0.0039	2	8	0.125	0.5
CIP101	GyrA D87N	2	0.25	0.125	0.03125	0.0625	2	8	0.0625	0.5

**Fig 3:**
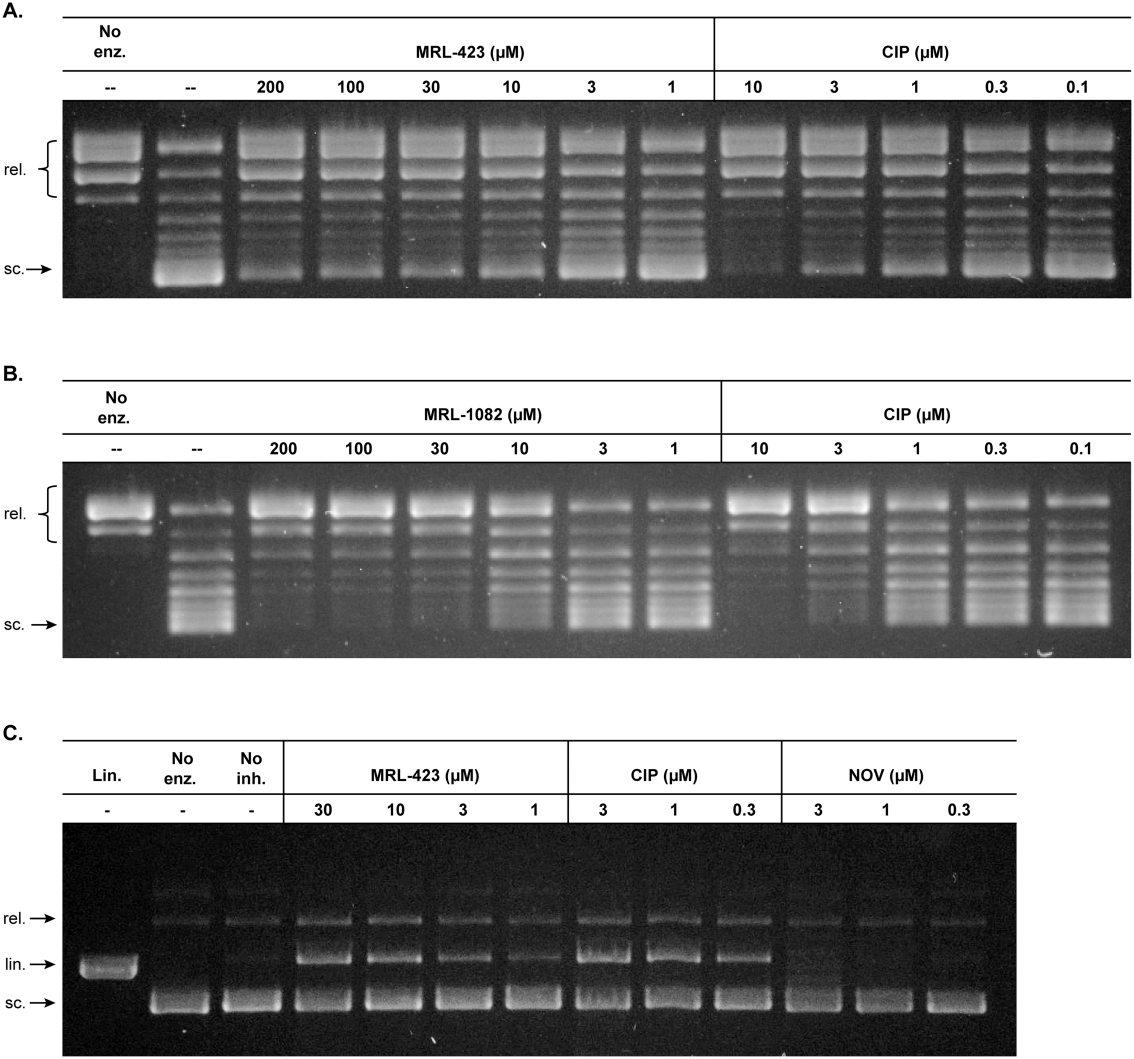
**Dose-dependent inhibition of *E*. *coli* (A) and *P*. *aeruginosa* (B) DNA gyrase by MRL-423 and MRL-1082 respectively**. (C) Dose-dependent stabilization of cleavage complex formation in *E*. *coli* DNA gyrase by MRL-423. Relaxed, closed circular substrate (rel.), linear (lin.), and super-coiled (sc.) DNA species are indicated to the left of each gel image.

The macromolecule synthesis profile and the identification of resistance mutations in a subunit of DNA gyrase strongly suggested that the initial hit molecule MRL-770 may be a DNA gyrase inhibitor. While the inhibition of *E*. *coli* DNA gyrase by MRL-770 is limited (S1 Fig) it did inspire us to make further modifications resulting in the synthesis of MRL-423 followed by MRL-1082. Fig 3A shows the results of a gel-based assay demonstrating inhibition of *E*. *coli* DNA gyrase by MRL-423 (IC_50_ ~ 7.5 μM). Consistent with the growth inhibitory activity of MRL-423 and MRL-1082 against efflux-defective *P*. *aeruginosa* (Table 1), MRL-1082 is active against *P*. *aeruginosa* DNA gyrase (Fig 3B). Our compounds did not exhibit any activity at the maximum achievable concentration (200 μM) against *E*. *coli* topoisomerase IV or *S*. *aureus* DNA gyrase (S2 Fig). With the improved antibacterial potency of MRL-423 and MRL-1082 we did observe relatively weak activity against mammalian cells in culture [41] at levels approximately 50-140-fold, respectively, above the MIC for *E*. *coli* HS151 (S1 Table). The mechanism of this cytological activity is unknown, but improvement in antibacterial activity of this series may expand that gap still further.

**Fig 4:**
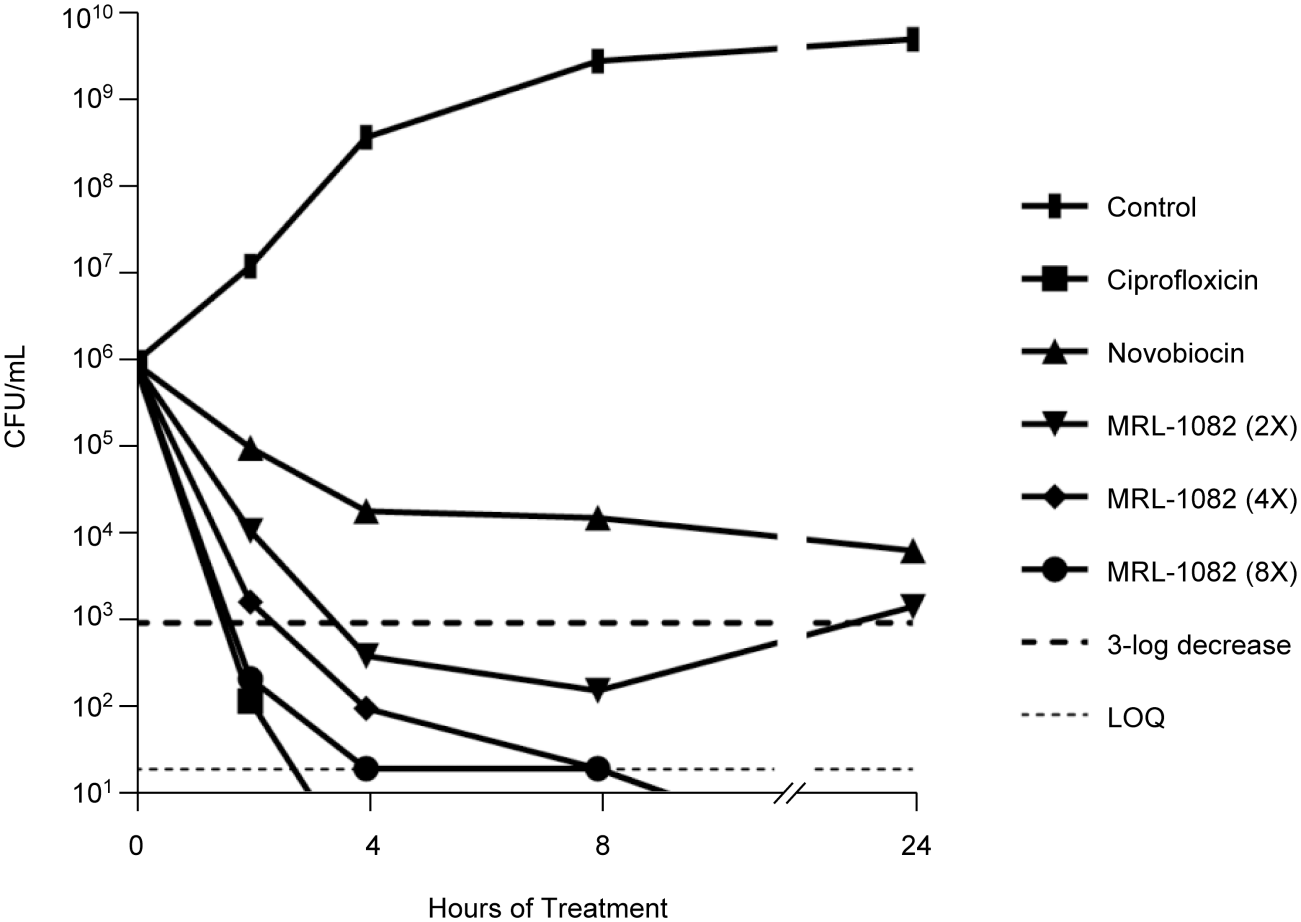
Time-dependent bactericidal growth inhibition by MRL-1082. *E*. *coli* HS151 was treated with ciprofloxacin (MIC = 0.00195 μg/mL), novobiocin (MIC = 2 μg/mL) or MRL-1082 (MIC = 0.0625 μg/mL) at the indicated concentrations. The ciprofloxacin treated culture was below the lower limit of quantitation (LOQ = 20 CFU/mL) at the 4 hour time point and the 4-8XMIC MRL-1082-treated cultures were below the LOQ after 8 hours. Isolates from the 2XMIC MRL-1082 treated culture at 24 hours were not resistant to the compound (MIC = 0.0625 μg/mL) suggesting that MRL-1082 had either deteriorated or precipitated to a level below the MIC.

Certain gyrase inhibitors, such as the FQs, poison gyrase in such a way that the double strand break in the substrate DNA is stabilized (cleavage complex) [42]. To explore the mechanism of inhibition of MRL-423 we tested whether the observed gyrase inhibition is accompanied by a similar blockage in the gyrase reaction cycle resulting in cleavage complex stabilization. As depicted in Fig 3C, like ciprofloxacin, MRL-423 stabilizes cleavage complex formation in a dose-dependent fashion. Cleavage complex stabilization by the FQs may be associated with the bactericidal action of this class [43]. Similar to the FQs and consistent with the notion that cleavage complex stabilization is a lethal event, this novel class of gyrase inhibitors shows potent bactericidal activity causing a greater than 3-log reduction in culture viability at MRL-1082 concentrations of 2XMIC or greater following 2–4 hours of treatment (Fig 4).

**Fig 5:**
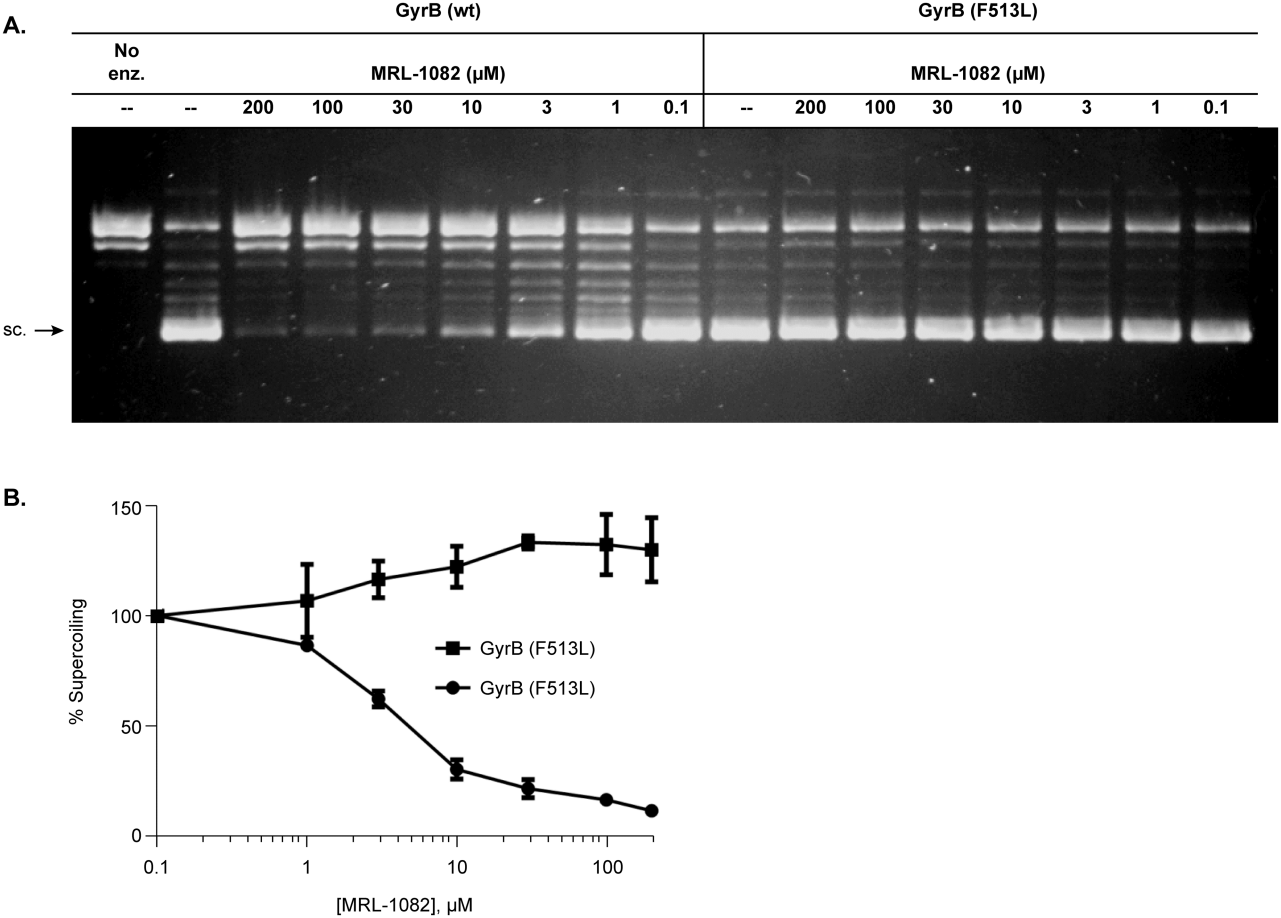
E. coli DNA gyrase GyrB (F513L) is resistant to inhibition by MRL-1082. (A) Agarose gel showing dose-dependent inhibition of wild-type *E*. *coli* DNA gyrase and resistance of DNA gyrase containing GyrB (F513L). (B) Quantitation of supercoiled product (sc). The 0.1 μM compound concentration served as the 100% control value.

An important prediction and test of the proposed mechanism of growth inhibition by these novel compounds is that DNA gyrase bearing a mutation conferring resistance in culture should also be resistant in vitro. To test the relationship between gyrase inhibition and bacterial growth inhibition DNA gyrase was reconstituted with either recombinant wild-type GyrB or GyrB (F513L) and inhibition by MRL-1082 was measured using the gel-based assay. We observed a significant difference in the sensitivity of the wild-type gyrase to induce supercoiling of the relaxed circular plasmid DNA substrate when compared to the GyrB (F513L) gyrase complex (Fig 5A) upon exposure to MRL-1082. Fig 5B depicts the quantitation of the supercoiled DNA in the gel indicating MRL-1082 dose-dependent decrease in supercoiling in the reaction containing the wild-type gyrase (IC_50_ ~ 3.5 μM), but not the GyrB (F513L) gyrase (IC_50_ > 200 μM).

Plasmid-encoded resistance to the FQs (*qnr*) takes the form of a small peptide that interacts with gyrase and protects it from inhibitor binding [44, 45]. Using a *qnr* determinant expressed from an *E*. *coli* plasmid (*qnrVC3*) [27], we tested whether this peptide can confer resistance to MRL-423. As shown in Table 3, *qnrVC3* expression conferred approximately 8-fold resistance to CIP in *E*. *coli* HS151 but had no effect on MRL-423 potency. Another protein known to interact with DNA gyrase is the chromosomally-encoded, Zn-binding YacG protein [46]. By binding to a specific region of DNA gyrase, remodeling a region of the complex, and blocking DNA binding, YacG selectively inhibits enzyme function [47]. An important residue in YacG that contributes to that binding and inhibition is YacG F40. The region of GyrB that YacG F40 interacts with is formed, in part by GyrB F513, a residue that has a role in sensitivity to our novel gyrase inhibitors. To test the contribution of YacG to the activity of MRL-423, we deleted *yacG* from the chromosome of *E*. *coli* BW25113 (Δ*tolC*) by allele replacement. As shown in Table 3, deletion of *yacG* does not affect the potency of MRL-423.

**Table 3:** Effect of a plasmid-based fluoroquinolone resistance determinant and gyrase regulatory factor YacG on activity of novel gyrase inhibitor.

compound	MIC (μg/mL)			
	HS151	HS151 (qnrVC)	BW25113 Δ*tolC*	BW25113 Δ*tolC*/Δ*yacG*
MRL-423	0.25	0.25	0.25	0.25
ciprofloxacin	0.004	0.03	0.004	0.004
novobiocin	2	2	2	2

## Discussion

The work described here originated from the systematic evaluation of a large set of small molecules with antibacterial activity. We applied established, classical methods such as macromolecule synthesis and resistant mutant selection and newer technologies likewhole-genome sequencing. As we refined this privileged set of bioactives we used these increasingly involved analyses to arrive at compounds with associated targets. One example is the novel gyrase inhibitor MRL-770. Taken together, the observations presented here demonstrate that we have discovered and improved upon a novel class of DNA gyrase inhibitors. These bactericidal compounds are active against efflux and permeability defective Gram-negatives and resistance arises at a very low frequency. While presently limited to Gram-negatives with impaired efflux or a permeability barrier defect, this class of compounds holds promise to treat isolates with FQ resistance arising from target site mutations and plasmid encoded *qnr* factors.

**Fig 6:**
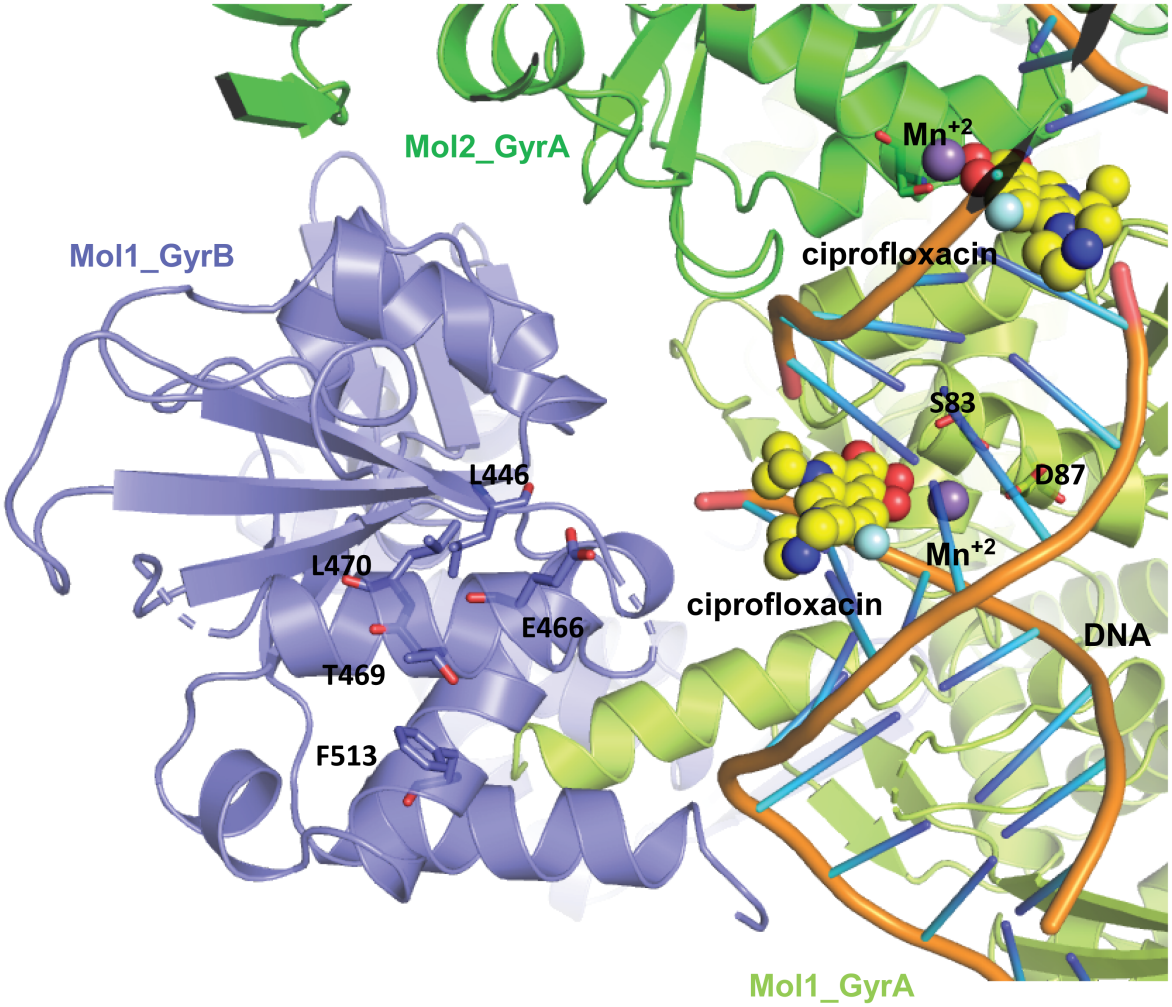
Mapping of the MRL-770/423/1082 resistance mutations onto a model of *E*. *coli* DNA gyrase (GyrBA fusion dimer) suggests a novel inhibitor interaction domain. Amino acids in the GyrB domain (light blue) where MRL-770/MRL-423 resistant primary mutations reside are rendered in stick form. The GyrA domains of monomers 1 and 2 are colored in light green and green respectively, S83 and N87 of GyrA monomer 1 are shown in stick form. The two ciprofloxacin molecules are displayed in CPK and carbon atoms colored in yellow. Nicked DNA is shown in orange and Mn^+2^ ions are in purple.

The X-ray structures of a number of bacterial type II topoisomerases have enabled a better understanding of the functioning of these molecular machines and aided in the discovery and refinement of enzyme inhibitors. The most elaborate structures comprise ternary complexes of gyrase, DNA substrate, and an inhibitor (e.g., [14, 48–50]). While we were not successful in obtaining a structure of our novel inhibitors in DNA gyrase, mapping of the amino acid residues in GyrB associated with resistance onto a model of DNA gyrase does provide some insights into the novelty and selectivity of these small molecules. To compare the sites of resistance with the binding of FQs and the relationship with the GyrA QRDR, we overlaid the *E*. *coli* DNA gyrase X-ray structure (PDB ID: 3NUH) [51] with a ciprofloxacin-bound *S*. *aureus* DNA gyrase X-ray structure (PDB ID: 2XCT) [14] and mapped the MRL-770/MRL-423 spontaneous resistance mutations onto GyrB (Fig 6). Most of the primary and reselected mutations reside adjacent to the ciprofloxacin binding site, but none appear to be in direct contact with ciprofloxacin. Glutamic acid 466 is the closest to the ciprofloxacin binding site (E477 in *S*. *aureus* DNA gyrase) at approximately 5 Å away. Interestingly, the only mutation we isolated at that site was a codon insertion conferring low level resistance to the FQs suggesting that an additional residue inserted at that site causes significant distortion of that region affecting binding of both FQs and our gyrase inhibitors. Additionally, cross-linking studies with FQ derivatives and *E*. *coli* DNA gyrase indicate a close juxtaposition of E466 and the C-7 pendant ring of ciprofloxacin [52]. GyrB F513 is the farthest away from the FQ/DNA binding region of any of the resistance mutations we isolated, but confers complete resistance to MRL-1082 at the cellular and enzyme level. This residue, F513, has been implicated in the binding of the gyrase regulator protein YacG (PDB ID: 4TMA) [47], but cells lacking YacG do not have altered sensitivity to our inhibitors. Two related compounds, QPT-1 and AZD0914 interact with the TOPRIM domain of *S*. *aureus* and *Neisseria gonorrhoeae* GyrB [17, 49], but mutations at sites conferring resistance to those inhibitors (*E*.*coli* GyrB G425, D426, and K447) are distinct from the sites conferring resistance to the inhibitors described here. With the close spatial association of the amino acid residues and the selective resistance observed with the primary and secondary mutations we isolated, the gyrase inhibitors we describe here appear to interact with DNA gyrase in a novel way.

The two analogs of the initial hit, MRL-770, were derived from an extensive structure activity relationship exploration. Medicinal chemistry efforts were focused on improving the overall physiochemical properties, including a reduction in clogP, in an attempt to improve cell potency. Details of that medicinal chemistry effort and the subsequent *in vitro* results will be the subject of a future publication. Further enhancement of antibacterial potency and mitigation of any mammalian toxicity will likely come from a better understanding of the interaction of this series with DNA gyrase and balancing that binding with enhancing the cell permeability characteristics of the molecule. Application of biophysical methods to quantitate compound uptake may allow a clearer understanding of the properties of this series that govern bacterial cell permeability [53, 54].

## Supporting information

S1 Fig The initial hit molecule MRL-770 inhibits *E*. *coli* DNA gyrase. Agarose gel showing pHOT-1 substrate (rel.) and reaction products (sc.) of *E*. *coli* DNA gyrase and the dose-dependent inhibition of enzyme activity by MRL-770 and CIP. Enzyme reactions and product analysis conducted as described in S1 Supporting information.
(TIF)

S2 Fig MRL-1082 does not inhibit *S*. *aureus* DNA gyrase (A) or *E*. *coli* topoisomerase IV (B). A. Agarose gel showing pHOT-1 substrate (rel.) and reaction products (sc.) of *S*. *aureus* DNA gyrase. B. Agarose gel showing substrate kinetoplast DNA (kDNA) and decatenated DNA (decat.) reaction products of *E*. *coli* topoisomerase IV. Enzyme reactions and product analysis conducted as described in S1 Supporting information.
(TIF)

S1 Table Mammalian cell cytotoxicity. (PDF)

S1 Supporting Information Supporting methods. *Staphylococcus aureus* DNA gyrase reaction and *E*. *coli* topoisomerase IV reactions.
(PDF)
