# Supplementary figures and images for: Antibacterial small molecules targeting the conserved TOPRIM domain of DNA gyrase
Source: PLoS One. 2017 Jul 10;12(7):e0180965. doi: 10.1371/journal.pone.0180965 (PMC5507300; doi:10.1371/journal.pone.0180965)

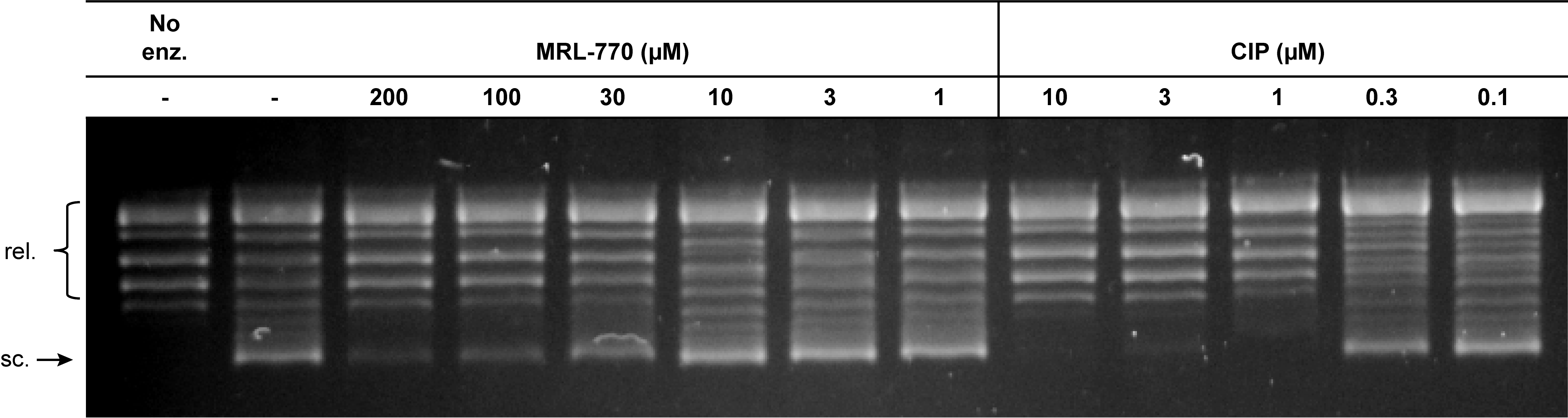

Supplement: S1 Fig — Agarose gel showing pHOT-1 substrate (rel.) and reaction products (sc.) of E. coli DNA gyrase and the dose-dependent inhibition of enzyme activity by MRL-770 and CIP. Enzyme reactions and product analysis conducted as described in S1 Supporting information. (TIF) [file pone.0180965.s001.tif]

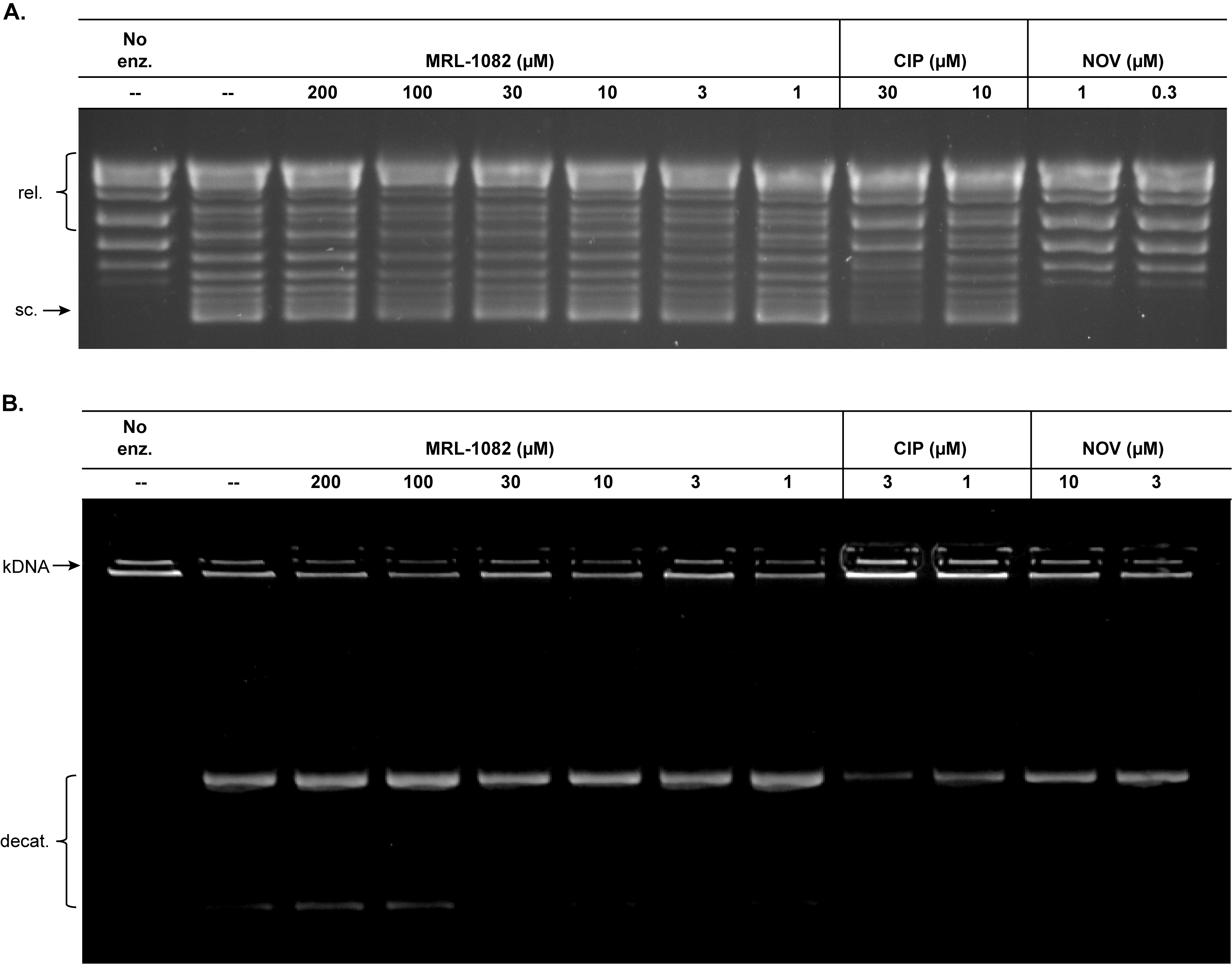

Supplement: S2 Fig — A. Agarose gel showing pHOT-1 substrate (rel.) and reaction products (sc.) of S. aureus DNA gyrase. B. Agarose gel showing substrate kinetoplast DNA (kDNA) and decatenated DNA (decat.) reaction products of E. coli topoisomerase IV. Enzyme reactions and product analysis conducted as described in S1 Supporting information. (TIF) [file pone.0180965.s002.tif]
