# Supplementary material for: Antibacterial small molecules targeting the conserved TOPRIM domain of DNA gyrase
Source: PLoS One. 2017 Jul 10;12(7):e0180965. doi: 10.1371/journal.pone.0180965 (PMC5507300; doi:10.1371/journal.pone.0180965)
Supplement: S1 Table — (PDF) [file pone.0180965.s003.pdf]

S1 Table: Mammalian cell cytotoxicity

	HeLa Cell EC50* (μM)	*E. coli* HS151 MIC (μM)	Fold above MIC
MRL-770	>100	4.5	>22
MRL-423	27.1	0.53	51
MRL-1082	18.2	0.13	140

*Average value (n = 2). HeLa cell cytotoxity determined as described in Mann, et al., ACS Infect. Dis. 2015, 1(1):58-72.
