# Supplementary material for: Antibacterial small molecules targeting the conserved TOPRIM domain of DNA gyrase
Source: PLoS One. 2017 Jul 10;12(7):e0180965. doi: 10.1371/journal.pone.0180965 (PMC5507300; doi:10.1371/journal.pone.0180965)
Supplement: S1 Supporting Information — Staphylococcus aureus DNA gyrase reaction and E. coli topoisomerase IV reactions. (PDF) [file pone.0180965.s004.pdf]

Supporting methods

***Staphylococcus aureus* DNA gyrase reaction:** Plasmid supercoiling reactions were assembled as suggested by the manufacturer of enzyme and reaction components (TopoGEN, Inc.) by combining 0.4 µL compound (in 100% DMSO)**,** 8 µL H_2O**,** 4 µL 5Xbuffer**,** 2 µL 10XATP solution, 0.5 µL relaxed pHOT-1 DNA substrate (0.5 µg)**,** 1 µL dilute *S. aureus* DNA gyrase (1U/reaction). Reactions were then extracted with 20 µL chloroform/isoamylOH (24:1) and a portion of the aqueous phase was loaded into a well in a 1% agarose/Tris-borate EDTA gel. Electrophoresis was conducted at 35 VDC until the loading dye migrated about 6.5 cm down the gel**.** DNA in the gel was then stained in 0.5 µg/mL ethidium bromide in water for 30 min. and rinsed (3X) in H_2O. Reactions were allowed to proceed for 30 min. at 37C and then stopped by adding 2 µL 10% SDS and 2 µL 0.5 mg/mL proteinase K (TopoGen). Following proteinase K reaction (30 min at 37C), 2.5 µL of 10X gel loading buffer were added and the mixture was then extracted once with 20 µL chloroform/isoamylOH (24:1) and a portion of the aqueous phase was loaded into a well in a 1% agarose/Tris-borate EDTA/ethidium bromide (0.5 µg/mL) gel and run at 50-65VDC until loading dye migrated about 6.5 cm down the gel. DNA in the gel was illuminated with UV light and photographed with an Alpha Imager (ProteinSimple, San Jose, CA).

***E. coli* Topoisomerase IV reaction:** To measure the decatenation activity of *E. coli* topoisomerase IV, reactions were assembled as follows using TopoGen, Inc. reagents: 0.4µL compound (in 100% DMSO), 4 µL 5Xbuffer (buffer A + B), 1 µL kDNA substrate (260 ng/reaction), 13.5 µL H2O, 1 µL topoisomerase IV (0.1 units/reaction, diluted in supplied dilution buffer). Reactions were allowed to proceed for 30 min. at 37C and then stopped by

adding 2 µL 10% SDS and 2 µL 0.5 mg/mL proteinase K (TopoGen). Following proteinase K reaction (30 min at 37C), 2.5 µL of 10X gel loading buffer were added and the mixture was then extracted once with 20 µL chloroform/isoamylOH (24:1) and a portion of the aqueous phase was loaded into a well in a 1% agarose/Tris-borate EDTA/ethidium bromide (0.5 µg/mL) gel and run at 50-65VDC until loading dye migrated about 6.5 cm down the gel. DNA in the gel was illuminated with UV light and photographed with an Alpha Imager (ProteinSimple, San Jose, CA).
